# What is critical for human-centered AI at work? – Toward an interdisciplinary theory

**DOI:** 10.3389/frai.2023.1257057

**Published:** 2023-10-27

**Authors:** Athanasios Mazarakis, Christian Bernhard-Skala, Martin Braun, Isabella Peters

**Affiliations:** ^1^ZBW – Leibniz Information Centre for Economics, Web Science, Kiel, Germany; ^2^Department of Organisation and Program Planning, German Institute for Adult Education - Leibniz Centre for Lifelong Learning, Bonn, Germany; ^3^Fraunhofer Institute for Industrial Engineering, User Experience, Stuttgart, Germany

**Keywords:** artificial intelligence, interdisciplinary, HCAI, human factors and ergonomics, information science, human-computer interaction (HCI), adult education, psychology

## Abstract

Human-centered artificial intelligence (HCAI) has gained momentum in the scientific discourse but still lacks clarity. In particular, disciplinary differences regarding the scope of HCAI have become apparent and were criticized, calling for a systematic mapping of conceptualizations—especially with regard to the work context. This article compares how human factors and ergonomics (HFE), psychology, human-computer interaction (HCI), information science, and adult education view HCAI and discusses their normative, theoretical, and methodological approaches toward HCAI, as well as the implications for research and practice. It will be argued that an interdisciplinary approach is critical for developing, transferring, and implementing HCAI at work. Additionally, it will be shown that the presented disciplines are well-suited for conceptualizing HCAI and bringing it into practice since they are united in one aspect: they all place the human being in the center of their theory and research. Many critical aspects for successful HCAI, as well as minimum fields of action, were further identified, such as human capability and controllability (HFE perspective), autonomy and trust (psychology and HCI perspective), learning and teaching designs across target groups (adult education perspective), as much as information behavior and information literacy (information science perspective). As such, the article lays the ground for a theory of human-centered interdisciplinary AI, i.e., the Synergistic Human-AI Symbiosis Theory (SHAST), whose conceptual framework and founding pillars will be introduced.

## 1. Introduction

The excitement around artificial intelligence (AI) is sparking a flurry of activity by researchers, developers, business leaders, and policy-makers worldwide. The promise of groundbreaking advances from machine learning and other algorithms drives discussions and attracts huge investments in, e.g., medical, manufacturing, and military innovations (Shneiderman, 2022). However, much of the debate in society is associated with aspects of whether or not AI will replace people in business activities (Del Giudice et al., 2023). In addition, trust in AI systems, transparency, and explaining such systems is not straightforward to end users (Laato et al., 2022).

In a survey in Germany in May 2023, 46% of 1,220 respondents considered AI technologies to be more of a risk for them personally, while only 39% saw opportunities in AI solutions. However, openness to these new technologies decreases with age and increases with education: most younger people up to age 34 see AI as an opportunity, as do individuals with a university degree (Infratest dimap, 2023). The results of previous surveys conducted worldwide are confirmed, although the results differ greatly depending on the economic development in each country (Ipsos, 2022).

More particularly, in the workplace, there is a risk of creating a defeatist mentality among the employees when ignoring human aspects of AI implementation. Similar examples exist from the past, e.g., knowledge management faced the same challenges around individual, organizational, and technological barriers (Riege, 2005). Nevertheless, it is argued that high levels of human control and automation are likely to simultaneously empower people and not just emulate humans (Shneiderman, 2020). The idea of human centeredness in AI implementation binds these critical research results together.

Artificial intelligence is, by definition, a sequence of mathematical models created by humans, which are executed by computers. The OECD defines AI more precisely: “An AI system is a machine-based system that can, for a given set of human-defined objectives, make predictions, recommendations, or decisions influencing real or virtual environments. [...] AI systems are designed to operate with varying levels of autonomy” (OECD., 2019, p. 23–24). This is a refinement of the definition of McCarthy (2007, p. 2), in which AI is defined as “[...] the science and engineering of making intelligent machines, especially intelligent computer programs. It is related to the similar task of using computers to understand human intelligence, but AI does not have to confine itself to methods that are biologically observable.”

In general, Artificial Narrow Intelligence (ANI) and Artificial General Intelligence (AGI) are distinguished. An AGI system would be an autonomous agent that can learn unsupervised (McLean et al., 2021). ANI has achieved enormous success in determined situations with a low-dimensional phase space, such as strategy games (Lenzen, 2019). However, the methodology of ANI performance can only be applied to a limited range of tasks (Landgrebe and Smith, 2022).

The organization of working processes may profit from a design approach that integrates human and technical intelligence. Such an organization is achieved, among other things, when humans and machines can use their specific skills and when humans and machines mutually support each other in gaining capabilities (Braun, 2017). In this sense, human-centered AI (HCAI) already consists of a set of standards, concepts, and principles, like e.g., fairness, accountability, beneficence, justice, and explicability, to name a few (Huchler et al., 2020). However, these principles are not consistently implemented in practice because of competing goals of productivity and cost-cutting, which has been a traditional challenge of HFE (Spitzley, 1980).

The present article aims at setting the foundations for an interdisciplinary theory of human-centered AI. For this, the article reflects in Section 6.2 on learnings from an interdisciplinary research project, which is considered as a demonstrator. This demonstrator combines normative, theoretical, and methodological concepts from human factors and ergonomics (HFE), psychology, human-computer interaction (HCI), information science, and adult education to study AI at work.

The article introduces each discipline's perspective on human-centricity and AI at work. It discusses the implications for developing, transferring, and implementing AI at the workplace, including disciplines not yet in the spotlight about HCAI at work. By explaining the fruitful interplay of these disciplines and how they contribute to human-centricity, we will ultimately argue that a theory of human-centered AI will immensely benefit from incorporating critical concepts, norms, and theories from the presented disciplines as well as from an interdisciplinary approach. This article, therefore, sheds light on different co-existing perspectives on and criteria of human-centered AI and will show how they can be meaningfully integrated to answer the research question of *what is critical for human-centered AI at work*. So far, this has only been conducted for, e.g., social sciences (Miller, 2019) and thus has not acknowledged or even included more diverse disciplines. However, many authors have identified an urgent need for collaboration across disciplines for human stakeholders, e.g., for explainable AI (Langer et al., 2021). This article contributes to this research gap by introducing views from heterogeneous disciplines with either a focus on individuals, such as psychology, or a focus on technology, such as HCI.

Moreover, the article leverages insights from disciplines that are either primarily concerned with a work context, such as HFE, or disciplines that study the work context as one research object amongst others, such as adult learning and information science. Furthermore, it systematizes results from the different disciplines by using the five perspectives on human-centricity by Wilkens et al. (2021). In addition, it identifies fields of action for human-centered AI implementation—such as supporting balanced workload, information literacy, providing tailored learning opportunities for low-skilled workers, enhancing technology acceptance, and building trust. By that, it finally sets the foundation for a synergistic human-AI symbiosis theory, which includes these aspects of AI implementation. Overall, this article is written for an interdisciplinary readership interested in human-centered AI. As a result, this article has more of an explorative, descriptive, and conceptual character.

## 2. Supporting balanced workload: HCAI in human factors and ergonomics

Human factors and ergonomics (HFE) has developed concepts and principles for work design, especially for human-technology interaction. These concepts can be applied to AI systems in terms of a division of functions between humans and AI. The principles of HFE inhibit a mutually reinforcing relationship between humans and AI based on capabilities and ethical design principles.

### 2.1. Defining ergonomics and illustrating the methodology

Ergonomics is the scientific discipline concerned with the understanding of interactions among humans and other elements of a system; ergonomics is also the profession that applies theory, principles, data, and methods to design in order to optimize human wellbeing and overall system performance (IEA, 2000). The terms *ergonomics* and *human factors* are often used interchangeably or as a unit (HFE). Domains of HFE are physical ergonomics (i.e., human anthropometric, physiological, and biomechanical characteristics as they relate to work), cognitive ergonomics (i.e., concerning mental processes), and organizational ergonomics (i.e., optimization of socio-technical systems, including their structures and processes). HFE is a multidisciplinary, human-centered integrating science that is not domain-specific (ILO and IEA, 2021). HFE encompasses not only safety and health but also the cognitive and psycho-social aspects at work. Additionally, HFE can focus on micro ergonomic design aspects—including the design of the procedures, the context, and the equipment and tools used to perform tasks—as well as macro ergonomic design aspects—including the work organization, types of jobs, technology, work roles, and communication (Wilson, 2014). Through their work activity, human beings acquire experiences about physical and social, external as well as internal reality, and they change by self-reflection (Schön, 1983).

A socio-technical “work system” is part of a work process in which a task is accomplished. Through the interaction of working humans with work equipment, the function of the system is fulfilled within the workspace and work environment under specific working conditions (ISO 6385, 2016). “Work design” is a collective term for measures for the purposeful and systematic design of work objects, work processes, and working conditions. The aim of work design is the optimal fulfillment of work tasks, taking into account human development requirements and economic efficiency (Dyckhoff, 2006). In order to increase productivity, work design is based on the rational principle of labor division and the systematization of work processes. The division of labor supports specialization, which makes it possible to use machines and automate work processes. Work design determines which functions are automated and which remain with humans (Baxter and Sommerville, 2011). The systematization of work sequences aims at their method-based optimization (Schlick et al., 2018). Since the division of labor goes along with external supply and a loss of autonomy, the social dimension of human-centered work seems to be indispensable; it is expressed, among other things, in committed cooperation, fair work relationships, and learning opportunities (Ulich, 2011).

### 2.2. Human-centered work design and interactive human-technology systems

Human-centered work design refers to a problem-solving approach from a human perspective and the interactions of the working human with technical and organizational factors. One application focus concerns interactive human-technology systems (i.e., interaction design). For this purpose, knowledge and methods from human factors, ergonomics, usability, and user experience are applied. Human-centered design criteria are documented in legal regulations, standards, and rules (Karwowski et al., 2021).

Human-centered work is characterized by balanced workload situations in order to avoid over-and under-strain and thus to promote performance, job satisfaction, and learning (Schlick et al., 2018). Work activities should not harm the health of the working person, should not-or at most temporarily-impair their wellbeing, meet their needs, enable individual and collective influence on working conditions and work systems, and contribute to the development of their personality in the sense of promoting their capability potentials and competencies (Ulich, 2011). Design dimensions include work content, time, process, conditions, workplace, or equipment.

Concerning the design of interactive human-technology systems, ISO 9241-2 (1992) Part 2 specifies the human-centered requirements of user orientation, variety, holism, meaningfulness, the scope for action, social support, and development opportunity. In ISO 9241-110 (2020) Part 110 (interaction principles), the human-centered design criteria of task appropriateness, self-describability, expectation conformity, learnability, controllability, error robustness, and user binding of technical systems are concretized. ISO 9241-210 (2019) Part 210 (Human-centered design for interactive systems) emphasizes the user experience, which describes the functional and emotional impressions of a user when interacting with a product or service. If users' requirements are met in a useful way, it is pleasant to use a product.

### 2.3. Human work and human factors

Technical rationalization measures aim to raise work productivity and reduce costs also by substituting the human workforce with machines. Such automation efforts can affect both physical and mental work. To automate sophisticated forms of work that were previously preserved by humans, the use of AI aims to imitate human intelligence (Landgrebe and Smith, 2022). In order to assess the extent to which AI can explain, predict, and influence human behavior requires HFE expertise.

Humans represent a physical, biological, social, and mental entity. They are generally aware that they have a perception, a mind, and a will. A basic requirement of human existence is to access the natural and social environment through interpretive understanding to gain meaning. In communicating with other humans and interacting with the environment, humans gain a deep understanding of the world (Bender and Kolle, 2020). On this basis, they can act purposefully even in the face of incomplete and contradictory information (Wilkens et al., 2014), transfer existing experiential knowledge to new contexts through the understanding of meaning, adopt different perspectives, and anticipate the consequences of their actions. However, the world can only be understood through knowledge of concepts and symbols that emanate from human intelligence (Whorf, 1963). Human intelligence is a complex neurological capacity including reasoning, memory, consciousness, emotions, will, intention, and moral judgment. Humans can take responsibility for acting solidaristic and morally (Böhle, 2009). Such subjective and implicit competencies are the basis of knowledge and innovation work:

- “Knowledge work” refers to the execution of work orders that are to be carried out according to available (un-) complete rules that the working person knows. Knowledge includes explicit or implicit components that guide action but cannot be verbalized.
- “Innovation work” refers to problem-finding and problem-solving work assignments in which the goal and the path to achieving the goal are not predetermined. Innovation work includes unplannable or poorly plannable, unpredictable intellectual performances, prognostic intellectual performances that do not have precisely defined goals, and diagnostic intellectual performances for which no algorithms can exist because it is unclear what is being searched for in the first place (Hacker, 2018).

There is increased interest to substitute knowledge work by AI. However, as expected, significant aspects of innovation work will remain with humans in the future (Hacker, 2018).

### 2.4. HFE design criteria for HCAI

HCAI encompasses individual safety, the trustworthiness of AI operation, an appropriate division of functions between humans and machines, and conducive working conditions (Huchler et al., 2020). Insofar as AI applications contribute to the automation of knowledge work, the design criteria of conduciveness to learning and social compatibility take on increased importance.

HCAI ties in with the human capabilities and places the mutual reinforcement of humans and AI at the center of their interaction. Such reinforcement will be reached through a complementary division of functions between humans and AI that takes into account the differences between human capabilities and technical functionalities (Rammert, 2009). The core principle of the complementary division of functions strives for higher productivity and adaptivity through a lower overall degree of automation with increasing partial automation and systematic integration of the capabilities of the working human (Huchler, 2022). In order to cope with the uncertainties of work systems, human options of control are extended, e.g., by informal work actions and work-integrated learning processes. Appropriate competencies are to be maintained by designing the interaction between humans and AI in a learner-friendly way (Grote et al., 2000).

Regarding the social responsibility of AI applications, ethical aspects need to be clarified. Ethics focuses on specifically moral action, especially with regard to its justifiability and reflection (Bostrom and Yudkowsky, 2013). In AI use, ethical questions are concretized in the phenomena of uncertainty and social inequality (Brynjolfsson and McAfee, 2012):

- *Uncertainty:* Purposes of the use of technology are not or not exclusively achieved, i.e., the relationship between means and ends is not always comprehensible; this manifests in insufficient transparency, comprehensibility, and manageability, as well as the irreversibility of decision-making processes.
- *Social inequality:* The people who suffer harm are not the beneficiaries; inequality affects personal and informational autonomy in the use of data, the possibility of personal development, decision-making power, and the economic exploitation of work results.

When designing HCAI systems, ethical rules should ensure that human autonomy of decision and action is preserved with moral intent (Floridi et al., 2018). Currently, AI is not yet comprehensively capable of making moral decisions. Moral principles are instead specified by humans and implemented in the form of algorithms that can lead to morally grounded actions as a result. An essential principle of human-centered design is preserving and appropriately using these moral means of control and access (Bülchmann, 2020). This also relates to the human influence of exit points, if necessary.

Bostrom and Yudkowsky (2013) recommend four ethical design principles of HCAI: An HCAI functioning should be (1) comprehensible and (2) its actions predictable in principle; there should be sufficient time for users to react and veto control in the event of a potential malfunction. HCAI should (3) not be easily manipulated, and if a malfunction does occur, (4) responsibility should be clearly defined.

HFE traditionally incorporates a variety of perspectives on human work. In this chapter, many methods and definitions of HFE were presented, which are also relevant to the other disciplines. HFE has a pragmatic concept for the design of work systems, processes, and tools, including AI, some of which are documented in regulations and standards. Knowledge of these methods and definitions is cross-disciplinary relevant, and not limited to HFE, and additionally necessary to prepare the implementation of HCAI at work.

## 3. Information is the key: HCAI in information science

The topic “human-centered AI” has not gained much attention in information science; even “human-centricity” is not a much-discussed concept due to how information science's central object of study — information — is conceptualized and defined. In the following, it will be argued that there is no need to be explicit about “human-centricity” because, for one, information does not exist outside of human beings, and second, information behavior is central to the development and evolution of humans. If human-centered AI systems are considered information systems, then several implications can be deduced for their design and handling from this argumentation. Overall, there are three ways how information science can conceptually approach human-centered AI:

1. The meta-level that discusses HCAI against the discipline's pragmatic understanding of information.
2. The information behavior perspective, which is considered central to human life and that leads to the creation of user models.
3. The literacy aspect that reflects on the skills humans need to handle AI successfully.

### 3.1. The meta-level: what is information?

In the literature, *information* is often characterized by using the Semiotic Triangle by Charles Sanders Peirce since it has been argued that information is the basis for the communicative action (in the sense of information as a message, information as a state) and a communicative act itself (in the sense of exchanging knowledge/being informed, as a process; Henrichs, 2014). Accordingly, information has a triadic structure consisting of the following:

- *Object*, meaning of the information or semantics,
- *Sign(s)/syntax*, signals for or physical carrier of knowledge and formal-syntactic representation of signs, and
- *Interpretant*, user/usage of information, or pragmatics.

This structure is dynamical since forming the relations between the parts always entails some sort of transmission. The structure is also relational, which results in the need to consider all three parts of the triangle simultaneously when referring to *information* since they are linked inseparably. Information science deals with all three aspects of information, leading to multi-and interdisciplinary studies with, e.g., computer science (that mostly focuses on the signal part, for example, when building digital libraries) or linguistics (focusing on the object part, for example when constructing ontologies or other knowledge organization systems). The most fruitful — and therefore most central — avenue for information science concerns, however, the pragmatics of information, which focuses on the human part of information processing and how humans make use of information. In a nutshell, information science seeks to understand how and for what reasons humans need, gather, and use information. It primarily asks from the interpretant point of view: What is information used for? Which actions are possible with that information, and what do humans need to act properly?

This understanding of information differs from the definition used in, e.g., computer science or telematics that focuses on the signal and disregards the meaning of the information (Shannon, 2001). Information science considers knowledge as the raw material for the creation of information — knowledge is possible information (Rauch, 1988), and information is a manifestation or representation of knowledge (Kuhlen, 2004). Since (formless) knowledge, which exists independently from signals, needs to be brought into a (physical) form to be transmitted (Stock and Stock, 2015), it can be argued that “information is a thing — knowledge is not” (Jones, 2010). If humans use that potential information for further action, information materializes. In general, information is used to decrease the amount of uncertainty a human experiences (Wersig, 1974).

Therefore, information depends on the context in which humans perceive it, and it can be different with different contexts and different humans. Humans construct information by decoding the signal — information does not just exist (whereas knowledge exists even without signals; Stock and Stock, 2015). This construction takes place in social environments and via means of communication. The recent popularity of ChatGPT and generative language models for AI, as well as how interaction (or communication) with those systems has been designed, is reflective of the relevance of communication in information processes and for information behavior.

### 3.2. Information behavior and user modeling: traces toward the human perspective

Reflections on the concept of *information* and studies on how humans engage with information have mutually influenced each other, developing a shared understanding of the subject. Information behavior research, as a sub-discipline of information science, is concerned with how and why humans interact with information in different informational contexts, including how they use, create, and seek information (Bates, 2017), actively or passively (Wilson, 2000), individually, collectively, or collaboratively (Reddy and Jansen, 2008). Information non-use, such as information avoidance (Golmann et al., 2017), is also part of the research agenda as well as information sharing, (personal) information management, information practices, information experiences, and information discovery (Greifeneder and Schlebbe, 2023).

It is remarkable that, similarly to the triadic structure that considers the human, the interpretant, an inseparable part of *information*, the concept of *Human Information Behavior* was never adopted by the research community (Bates, 2017). Information behavior research underwent several so-called conceptual and methodological turns that are also reflective of the increasing relevance and attention the human being has been attributed over the years: from understanding which information sources and systems humans use to gather information, learning about the information need that motivates humans to interact with information (cognitive turn) and their emotions involved (affective turn), to the role of socio-cultural contexts (socio-cognitive turn) and habitualized information practices (social-constructionist turn) (Hartel, 2019). Information behavior can only be exposed by humans — manifesting their (for outsiders' implicit) relevance for information science again.

This also becomes apparent by one central activity of information behavior, i.e., the humans' engagement in looking for information. Case and Given (2016) emphasize that from birth onwards, humans are prompted to seek information to meet their fundamental needs. Information needs are driven by those fundamental needs, often because the human recognizes a lack of information to meet the fundamental needs (Stock and Stock, 2015). Information seeking behavior is activated by concrete information needs and is, therefore, active and intentional (Case and Given, 2016). If a computer or IT system is used to look for information, then Wilson (1999) speaks of Information Searching.

Information behavior research is not a goal in itself — like other disciplines, it seeks to advance information systems to tools that can be easily and efficiently used, that automatically adapt to changing situations, and that are adaptable to the needs of their users (Elbeshausen, 2023; Lewandowski and Womser-Hacker, 2023). As has been argued, understanding information and information behavior always requires knowledge about humans, e.g., users of an AI system. The complexity of information behavior often prevents the use of quantitative or statistical methods, so that qualitative methods are the main approach.

User modeling is an important activity in this regard, which aims to describe individual users to enable, for example, personalization of search results or groups of typical users that share certain characteristics (e.g., novices and experts). Latter is often realized via personas that represent typical users of a system with very concrete properties (an approach sometimes criticized for replicating stereotypes; Marsden and Haag, 2016). If the information system targets a broad user base, user modeling can be a tough challenge since there is not only a large, diverse group of (possible) users, but user behavior is also dynamic and can change while using the system for particular tasks or over time. Humans adjust informational practices, tactics, and behavior dynamically to match contexts and to maximize the amount of information they can get, e.g., by changing search terms (Pirolli and Card, 1999). In addition to the informational environment and contexts, the information behavior of a person is also connected to their personality (Lewandowski and Womser-Hacker, 2023). The principle of least effort (Zipf, 1949) is also applicable to information behavior: humans tend only to spend the minimum effort to accomplish tasks, often only resulting in only a satisfying (but not the best) result.

Interactive information systems that more dynamically react to users' information behavior and that serve a variety of tasks, need to even better understand humans, their needs, and context to be accepted and add value. Ingwersen and Järvelin (2005) argue — similar to the pragmatic definition of information — that information systems are never used in isolation but are always embedded in personal, organizational, cultural, and more contexts and therefore need to be designed and evaluated within those contexts. Users should be given the opportunity to use information systems purposefully to focus on the task to be fulfilled without being bothered by the challenges of handling the system (Elbeshausen, 2023, p. 474). For economic reasons, in the corporate context, it is of paramount importance to know which information types and information services are meaningful for employees and which information needs arise (Stock and Stock, 2015). Gust von Loh (2008) distinguishes between objective information needs from workers and employees that arise from a certain job position (e.g., because of the company strategy) and that are independent of a particular staff member, and subjective information needs that are articulated by a specific job holder and that stem from user studies.

Here, strong connections to the human-computer interaction (HCI) field become apparent. Information science and HCI share their focus on humans interacting with information systems, their cognitive and contextual embeddedness while doing so, and the subjective and objective information needs a system has to satisfy (Jetter, 2023). Both disciplines acknowledge that the design of humane (or human-centered) information systems (empirically proven via usability-and user experience methods) benefits from the enrichment of information and contextualization.

Technical information systems may be unable to fit all the information behavioral aspects of a broad user base but may need to focus on a selection of tasks or user types. Furthermore, information behavior also takes place outside of technical or digital environments. Then, educating the users toward a certain behavior and increasing their knowledge about the information system and environment could be an additional approach.

### 3.3. Information literacy as a prerequisite to deal with AI

Although information is central to human development and life, dealing with information in a good and meaningful way is a skill that has to be acquired and cultivated — especially with regard to the ever-increasing complexity of today's digital information environments. To be able to efficiently and ethically deal with information in a particular context, to understand how information is produced, evaluated, and distributed, how it can be effectively searched for, and to assess the personal informational and thinking competencies critically are skills that are subsumed under the term “information literacy” (Griesbaum, 2023). The UNESCO (2013) considers media and information literacy as a core competency for democratic societies that enables citizens to successfully engage and participate in private, vocational, and societal activities. Information literacy is, however — and similarly as the concept at its core: information — a relational concept. Its characteristics change with the information environments and contexts in which human beings have to deal with information (Griesbaum, 2023). This also presumes that an information-literate person has a certain amount of knowledge about the topic or circumstances they are dealing with — but this is not always the case. Hence, the more the person lacks expertise and knowledge, the more trust the person needs to put into the information ecosystem. Information literacy then transfers from the topic or situation itself to the evaluation of other information sources or experts whose recommendations have to be trusted (Griesbaum, 2023).

Despite its stated relevance, often, information literacy is not an integral part of school education but rather embedded in higher education and services of university libraries (ACRL, 2016). In work-related contexts, information literacy issues become apparent in enterprises with structured knowledge management approaches (Travis, 2017). However, Lloyd (2013) has found that information literacy at the workplace is mainly reduced to socio-cultural practices for collaboration. Middleton et al. (2018) could show that information literacy is strongly connected to innovative work practices.

This hints toward an increasing need for information literacy in complex information contexts as induced by AI systems. Consequently, AI literacy is an emerging field in information science, borrowing most of the central aspects already embedded in information literacy but also highlighting further skills and normative claims (Touretzky et al., 2019). Ng et al. (2021a,b) performed a literature search on AI literacy to derive aspects this concept entails. They found that all selected articles consider knowing the basic functions of AI and how to use AI applications in everyday life ethically (know and understand AI), as well as applying AI knowledge, concepts, and applications in different scenarios (apply AI), the core competencies of AI-literate humans. Two-thirds of the analyzed articles also mention critical higher-order thinking skills (such as evaluating, appraising, predicting, and designing) as part of AI literacy (evaluate and create AI).

Furthermore, the literature states that AI literacy is central to the future workforce, simultaneously preparing humans to efficiently use and critically evaluate AI and sparking career interest in this field (Chai et al., 2020). Interestingly, the study revealed that only 50% of the articles considered educating humans about socially responsible behavior when using or designing AI as part of AI literacy. Here the authors see room for improvement: “[…] conceptualizing AI literacy with human-centered considerations is crucial to building a future inclusive society” (Ng et al., 2021a, p. 507). The evaluation of AI literacy itself is conducted via knowledge tests, self-reporting, questionnaires, or observations when interacting with AI.

## 4. Designing learning opportunities for all: HCAI in adult education research and adult learning

The core assumption of HFE is that human beings are able and willing to learn and shape their working lives. Considering this first assumption, aspects of lifelong learning, adult and continuing education, and adult learning at the workplace touch the core of work design and human centeredness. At the same time, for many years adult education policies and research have dealt with how educational systems can effectively provide knowledge and skills for a technologically changing (working) society (Merriam and Bierema, 2013). The results of numerous research activities within the adult education scientific community contribute to shaping AI-affected workplaces in a human-centered way, which from an adult educational perspective means a learning-centered way (Harteis, 2022).

From the perspective of adult education research and policy, it is a consensus that educational systems target fostering the quality of educational processes and providing learning opportunities equally (UNESCO, 2019; BMAS and BMBF, 2021; Council of the European Union, 2021; Autor:innengruppe Bildungsberichterstattung, 2022; OECD, 2023).

Educational systems contribute to designing and establishing *learning opportunities*. That means they contribute to channeling, organizing, and monitoring informal, non-formal, and formal (adult) learning processes. In democratic states, educational systems aim to provide skills to individuals so that they can actively participate in public and working life. At the same time, quality learning processes within educational systems underlie the expectancy to provide a qualified and employable workforce. Scientific discourse treats these aspects using the two concepts of individual self-regulation, on the one hand, and human resources, on the other hand (Autor:innengruppe Bildungsberichterstattung, 2022).

Work has a major role to play in education. First, work and working life are significant fields of adult learning. Human subjects acquire skills for and within their employment to stay employable. Second, in Western-so-called working societies-work is a major part of active participation in society, as it impacts, e.g., social status and social and financial resources as much as professional and, therefore, social identity (Kraus, 2008; Gericke, 2017). Third, work in terms of work-based learning is a learning and teaching methodology (Bauer et al., 2004; Dehnbostel, 2022). Therefore, it is an important quality criterion of professionally designed adult and continuing education to take aspects of individuals' (working) lives, such as possible ruptures in (working) biographies and career development, as one starting point for developing and creating learning opportunities.

*Equality of opportunities* in adult education refers to equal access to learning opportunities as a major challenge for education systems (Käpplinger and Lichte, 2020; Council of the European Union, 2021). It targets especially vulnerable and marginalized groups such as migrants, low-qualified, unemployed, disabled, or illiterate persons, who have different learning needs regarding content but also need differently structured learning opportunities than high-skilled workers or middle-class citizens. Work has an important role to play in the equality of learning opportunities. According to the Adult Education Survey (BMBF, 2022), for years, more than 70 up to 75% of the adult learning activities of the German population aged 16–65 have taken place during daily working time or were financed by the employer. A much smaller and even decreasing part of adult learning activities was work-related but based on individually generated financial and time resources (13% in 2012 – 8% in 2020), while the share of individual non-work related learning activities is relatively stable at about 17–18% (BMBF, 2022, p. 22). Major differences exist in the participation rates of different social groups. The employed population shows a higher participation rate (46% in 2012 – 60% in 2020) than the unemployed population (13% in 2012 – 19% in 2020). The same applies when comparing the un-or low-skilled population (30% in 2012 – 46% in 2020) with high-qualified persons (about 70% in 2012 – 81% in 2020). Remarkably, in the German adult population, learning activities are on the rise in absolute numbers. At the same time, the differences in share between certain social groups have not remarkably diminished. These findings concerning the participation rates in adult learning vary across countries, still the gap between employed and unemployed, as much the high – and the low-skilled persons, shows to be a central challenge in more or less all OECD countries (European Union, 2021).

Against this background, an adult education research perspective on human-centered AI implementation will concentrate not only on how to provide quality learning opportunities but it will focus as well on how to tailor these quality opportunities for each social group. So, an adult education research perspective contributes to human-centered AI, first of all, by analyzing if an educational system, an employer, or a single workplace offers learning opportunities for AI-based workplaces, whom these learning opportunities are made for, and what kind of learning opportunities are proposed.

### 4.1. Designing learning: what skills should we qualify for?

Regarding contents and needs for skills, there is consensus that in a digitalized and AI-based world, life and work tasks will become more complex. There are catalogs trying to capture and describe important future skills. In the context of education and lifelong learning, the European Commission's Framework DigComp has had quite an impact in the field in Germany (Joint Research Center (JRC), 2022). Moreover, in higher education, the so-called twenty first-century skills play an important role (Anandiadou and Claro, 2009; Schnabel, 2017). These two catalogs represent important examples for a whole discussion that brings the importance of future skills to the fore. They concentrate on skills in

- Working with media, technology, information, and data
- Virtual and face-to-face communication and collaboration in diverse (e.g., interdisciplinary, intercultural, intergenerational) contexts
- Creative problem solving, innovation, analytical and critical thought
- Flexibility, coping with ambiguity, self-motivation, and working independently (Schnabel, 2017)

When thinking human centeredness from an adult learning perspective, it is important to note that these skills will not replace professional skills but will additionally be on top of professional skills. Even more, they will be interlinked with professional skills. So when preparing a workforce for an AI working world, degrees will have to encompass professional skills as a basis plus these future skills.

### 4.2. Designing learning: who should we target and how?

Quality is not determined by knowing the skill needs and contents of learning but also by methodologies that help to teach these skills professionally, effectively, and efficiently to a whole range of target groups. When it comes to skill delivery in companies, there is a vivid research landscape on how to deliver sustainable learning success in digital transformation. The learning and teaching methodologies in focus range from informal learning in the workplace, learning nuggets, non-formal workshop settings, or formal learning arrangements within chambers and universities (e.g., Rohs and Ganz, 2015; Anderson and Rivera-Vargas, 2020). From the company's viewpoint, where there are financial and economic restrictions, these discussions are critical. With a company's decision to invest in one or another kind of learning opportunity, it shapes structures and methodologies of learning and, finally, participation rates in adult learning opportunities to a high degree.

Discussions of teaching and learning methodology differentiate along the question of which knowledge or skill can be efficiently and professionally taught in which setting to which target group. Taking marginalized groups as an example, it is a common educational argument based on Bourdieu (Watkins and Tisdell, 2006) or biographical research (Alheit, 2021) that low-skilled people or functional illiterates have rather negatively experienced learning throughout their lives, sometimes they have gone through biographies of failing in an educational system. Therefore, it is highly challenging for professionally organized adult education to get access to these groups and to teach them effectively-much more challenging than teaching high-skilled people or managers who have had successful learning careers.

Therefore, when implementing AI in a human-centered way, quality learning opportunities need to ensure that all target groups who are affected by AI in the workplace get the opportunity to qualify for these changes. At the same time, different target groups will need different skills in the workplace and different learning methodologies for acquiring these skills. In addition, it is a professional adult education task to create good learning opportunities with a well-fitted methodology that facilitates between the affordances of a company within the digital transformation, on the one hand, and the needs of the target group and their learning habits, on the other hand.

In terms of learning methodologies, recent projects have shown

(a) That professionally implemented learning projects in the workplace can effectively qualify low-skilled workers on the job within digital transformation processes (Goppold and Frenz, 2020).
(b) That worker's councils have an important role to play as facilitators of bringing together unskilled and low-skilled workers or functionally illiterate employees with continuing education activities. Still, the members of worker's councils need to be qualified to fulfill their role (Lammers et al., 2022; Arbeiter, 2023).

In recent years, networked structures in adult education have been brought to the fore (e.g., UNESCO, 2015). In the case of implementing human-centered AI networks between adult education providers, companies and worker's councils will probably be in favor of channeling professionally tailored learning opportunities into companies. Taking Germany as an example, vocational training providers create those networks in order to target marginalized groups; in the case of high-skilled individuals, universities of applied sciences have a mandate of developing continuing education to create opportunities in cooperation with companies (Dollhausen and Lattke, 2020). It is an issue if these networks allow scaling up learning opportunities for a whole population or multiple companies and not to tailor adult learning for one single company.

## 5. Technology, autonomy, and trust: HCAI in psychology and human-computer interaction

Typically, the AI research community focuses on algorithmic advances, deeming a human-centered approach unnecessary, but at the same time, human-centered thinking is gaining popularity, and the AI community is diverse. However, this new thinking challenges established practices (Shneiderman, 2022, p. 40). This new thinking also influences the perception of psychology and HCI, which are closely related, although they are separate disciplines, because psychology plays a significant role in HCI. For this reason, both disciplines are discussed together in this section (Clemmensen, 2006). From a psychological and human-computer interaction (HCI) point of view, technology acceptance and adoption are also becoming essential aspects of human-centered AI (Del Giudice et al., 2023), especially in human autonomy (Bennett et al., 2023) and the development of guidelines for human-AI interaction (Amershi et al., 2019). Such guidelines need to consider issues with information overflow and should assist in using complex systems (Höök, 2000). This consideration can be achieved by putting in place verification measures or regulating levels of human-controlled autonomy to prevent unintended adaptations or activities by intelligent systems (Amershi et al., 2019; Xu et al., 2023). In addition, AI-driven influence techniques like psychological targeting or digital nudging have raised ethical worries about undermining autonomy (Bermúdez et al., 2023). Moreover, a series of recent studies found that employees who work with AI systems are more likely to suffer loneliness, which can lead to sleeplessness and increased drinking after work (Tang et al., 2023).

Nevertheless, in HCI, the understanding of human autonomy remains ambiguous (Bennett et al., 2023). This ambiguity might be attributed to an old controversy if people and computers being in the same category or if, as many HCAI sympathizers believe, vast differences exist (Shneiderman, 2022, p. 25), with Shneiderman supporting the latter (Shneiderman, 2022, p. 31). However, AI and its impact on the workplace are said to be disruptive, including chatbot-based communication systems that can demonstrate empathy through an understanding of human behavior and psychology, allowing the chatbot to connect with customers emotionally to ensure their satisfaction and thus support the adoption of AI systems (Krishnan et al., 2022). AI differs from HCAI by two key human-centered aspects in terms of performance and the product. The human-centered process is based on user experience design methods and continuous human performance evaluation. Furthermore, the human-centered product is emphasized by human control to enhance human performance by designing super tools with a high level of automation (Shneiderman, 2022, p. 9).

Still, HCI acknowledges the importance of AI by highlighting it in almost all of the current HCI grand challenges, like human-technology symbiosis and human-environment interactions, to name a few (Stephanidis et al., 2019). This is accompanied by six grand challenges of human-centered AI: human wellbeing, responsible design of AI, privacy aspects, AI-related design and evaluation frameworks, the role of government and independent oversight, and finally, HCAI interaction in general (Garibay et al., 2023). HCAI interaction especially plays a vital role at work, as economic challenges meet with ethical and organizational considerations (Garibay et al., 2023). This collection of grand challenges reflects the almost symbiotic relationship between HCI and AI.

Finally, the transition to human interaction with AI systems by moving on from siloed machine intelligence to human-controlled hybrid intelligence can be considered a new opportunity for HCI professionals to enable HCAI (Xu et al., 2023). A potential goal of human-centered AI design is to create human-controlled AI using human-machine hybrid intelligence, which emphasizes the integration of humans and machines as a system, aided by the introduction of human functions and roles that ensure human control of the system (Xu et al., 2023, p. 503). However, such integration of humans and machines is not without obstacles and unrealistic user expectations, and negative emotional responses are often a source of concern.

### 5.1. Unrealistic user expectations

Exaggerated and unrealistic user expectations about AI-based applications and absent design solutions to support human-centered work can lead to frustration and questioning the “intelligence” of such applications (Luger and Sellen, 2016). For example, high efficiency of search functions should be combined with curated content and meaningful recommendations even without the necessary meta-information. These demands raise hopes that may neglect the actual software and hardware capabilities of research projects, which may only be feasible for very large companies. In addition, it is requested to combine, match, and recommend different kinds of heterogeneous data, even on the internet, without considering resources. Although AI makes significant improvements daily, these are still quite unrealistic user expectations today.

One possible way to overcome these challenges of unrealistic user expectations is to divide the AI-based processes into different phases. For example, Amershi et al. (2019) offer 18 AI design guidelines separated into four phases: the initial phase when beginning to work with an AI-based application, during general interaction, when things go wrong, and aspects considering long-term experiences. A vital aspect of these guidelines is providing support and managing expectations. Such aspects are not unknown to technology acceptance models.

### 5.2. Building on technology acceptance and trust

Technology acceptance models, e.g., TAM (Davis, 1989), UTAUT (Venkatesh et al., 2003), and their extensions in various fields (Kao and Huang, 2023), offer a promising domain for an evaluation concerning human-centered AI. TAM is a conceptual model used to account for technology usage behavior, which has been confirmed to be valid in various technologies among different groups of people (Venkatesh et al., 2003; Choung et al., 2023). The original TAM model postulates that the intention to use technology in the future is determined by two key factors: perceived usefulness and perceived ease of use (Davis, 1989).

Choung et al. (2023) integrated trust as an additional variable in their extended TAM model. Their two studies confirm that trust is vital for accepting technology. Therefore, AI technologies should be designed and implemented in a human-centered way; consequently, their implementation should be easy to use, useful, and trusted. In general, empirical findings support the assumption that technology acceptance models help to explain the acceptance of AI technologies (Sohn and Kwon, 2020), including the aspect of trust (Choung et al., 2023). However, nevertheless, there are limitations to their usage, which are discussed by Bagozzi (2007).

Users' low level of trust in how their data is handled and processed must also be adequately considered psychologically. AI, in general, can predict user behavior in a wide range of applications by following digital traces of usage. Besides legal and ethical challenges, psychologists call this approach digital phenotyping when using elaborated smart sensing techniques and when it is successfully assisted and analyzed by data mining and machine learning tools (Baumeister et al., 2023). This is not an entirely new topic, as, e.g., user behavior in an online environment relates to their personality and can be used to tailor content, improve search results, and increase the effectiveness of online advertising (Kosinski et al., 2014), which is backed by many empirical studies and summarized by Baumeister et al. (2023). At the same time, ethical challenges are addressed by the human-in-the-loop design, where individuals are asked to make a final decision or action (Shneiderman, 2022; Garibay et al., 2023; Xu et al., 2023), which can also help to improve the trust to HCAI.

This is in line with results from an experiment by Westphal et al. (2023), in which they empower users to adjust the recommendations of human-AI collaboration systems and offer explanations for the reasoning of the systems. The idea behind this approach is to counter low trust and limited understanding of users dealing with recommendations of an AI system, and at the same time, to keep in mind to achieve an adequate or calibrated trust, meaning that, e.g., not to over trust the AI system (Leichtmann et al., 2023b). However, interestingly, explanations could backfire because they can increase or signal task complexity, whereas enhanced decision control leads to higher user compliance with system recommendations (Westphal et al., 2023). These results affirm that well-explained support can be essential to accept and facilitate HCAI at work, leading to HCAI systems that explain themselves, so-called human-centered explainable AI, which can be accompanied by educational offers and measures for providing human-centered explainable AI.

### 5.3. A glimpse into the near future: explainable, understandable, and gamified AI

Such explainable AI, especially if it is human-centered, can be considered crucial. However, from a socio-technical standpoint, AI should also be understandable to stakeholders beyond explainability (Habayeb, 2022). This can be achieved when implementing user-participated experimental evaluation because it is necessary to overcome the relatively simple unilateral evaluation methods that only evaluate AI systems' performance.

One way to implement this is to use, e.g., a gamified crowdsourcing framework for explainability (Tocchetti et al., 2022), which uses game design elements in a non-game context (Deterding et al., 2011). However, current research focuses primarily on strategic and system issues related to AI system performance (Raftopoulos and Hamari, 2023), which limits the view of AI. Furthermore, HCI should promote the evaluation of AI systems as human-machine systems by including the end-user perspective (Xu et al., 2023, p. 505). Nevertheless, this makes it necessary for HCI to enhance its current methods. Constraints like focusing on single user-computing artifacts with a limited context of use, lab-based studies, or static human-machine functions are prevalent. Instead, “in-the-wild studies,” the application of distributed contexts of use, and longitudinal study designs are encouraged to address the identified unique issues of AI systems to influence the development of AI systems in a human-centered way (Xu et al., 2023, p. 509–512).

Additionally, incentives for using artificial intelligence, e.g., gamification, are also emerging topics of interest for human-centered AI (Mazarakis, 2021). Gamification tries to bring the motivating effect associated with games to non-game situations with the help of elements like badges, leaderboards, and points (Mazarakis, 2021, p. 279, 283). First studies conducted with intelligent user interfaces and voice user interfaces like Amazon Alexa, which are also considered social robots and active appliances in artificial intelligence (Shneiderman, 2022), show the potential to focus on empirical research for the acceptance of these interfaces (Bräuer and Mazarakis, 2022a,b; Haghighat et al., 2023). Nevertheless, explainable AI is also, in this use-case, key to counteracting suspicion regarding the trust of social robots and active appliances in artificial intelligence. For example, to achieve transparent and accountable conversational AI and to include such a system in a gamified environment, interpretability, inherent capability to explain, independent data, interactive learning, and inquisitiveness are necessary (Wahde and Virgolin, 2023, p. 1856). Inquisitiveness is meant to be by the AI to show curiosity and not to annoy the user to achieve human centeredness. Curiosity means just displaying inquisitiveness in specific contexts, such as during learning, so as not to disturb the user (Wahde and Virgolin, 2023, p. 1865).

A further step is taken by Tocchetti et al. (2022), which propose a gamified crowdsourcing framework for explainability. Their crowdsourcing framework engages users on different levels than other platforms, primarily relying on extrinsic rewards. The provided user education, in particular, would raise users' understanding of the types of information that an AI system requires, learns, and produces, improving users' efficiency and developing the users' mindsets (Tocchetti et al., 2022, p. 7). Furthermore, their work shows that a symbiosis of HCAI, gamification, and explainable AI is also possible with greater effort and exertion. Consequently, this also results in an increased human centeredness. A first effort of studies in game-based environments yields promising results for explainable AI (Leichtmann et al., 2023a).

Different scenarios for using gamified AI and gamification in the context of AI, in general, are possible. For example, Tan and Cheah (2021) describe a work in progress and prototype for developing an AI-enabled online learning application for lecturing at a university physics. However, this scenario can be switched to a work-related setting without much effort. As education is one of the main application areas of gamification (Mazarakis, 2021), a combination with AI is obvious and already taking place (Kurni et al., 2023). In this case, first data is collected for AI processes, e.g., through step-by-step scaffolding instructions and feedback to students by studying students' progress in answering quiz questions. Then, it is possible to implement adaptive assessments to more accurately identify the student's level of mastery, adjust the difficulty level and the number of questions at each level of difficulty, and finally, step between each level of progression based on the student's answer. Thereby, individual feedback, which can then be used for learning analytics to improve, optimize, or redesign the curriculum to meet the needs of specific student cohorts, can be provided (Mazarakis, 2013; Tan and Cheah, 2021), e.g., for employer-provided training in different work scenarios.

## 6. Discussion

This chapter presents conclusions from the previous chapters' theory and practice and shows relations between them in order to inform a synergistic human-centered AI theory. First interdisciplinary human-centered AI perspectives are shown, according to Wilkens et al. (2021), and how they relate to the five disciplines. Then, interdisciplinary views of human-centricity and their interrelations are matched with observations from a demonstrator. These views have the goal of setting foundations for an interdisciplinary synergistic theory of human-centered AI, which is presented in Section 6.3.

### 6.1. Interdisciplinary perspectives on human-centered AI

Wilkens et al. (2021) found five co-existing views in a comprehensive literature review analyzing the significance of HCAI: a deficit-oriented, a data reliability-oriented, a protection-oriented, a potential-oriented, and a political-oriented understanding of how to achieve human-centricity while deploying AI in the workplace. These five perspectives reflect many aspects of AI's human-centricity, with varying levels of maturity along each dimension. In order to put the results of this article into context, the disciplines of information science, human-computer interaction, psychology, and adult learning are related in Table 1 to the five perspectives of Wilkens et al. (2021). HFE is inherent in Wilkens et al. (2021) and would cover all five perspectives, so it is not shown in Table 1.

**Table 1:** Analysis of human-centered AI perspectives for information science, adult education, human-computer interaction, and psychology according to Wilkens et al. (2021).

**Discipline**	**Deficit-oriented (Perspective 1)**	**Data reliability-oriented (Perspective 2)**	**Protection-oriented (Perspective 3)**	**Potential-oriented (Perspective 4)**	**Political-oriented (Perspective 5)**
Information science			x	x	x
Adult education			x	x	x
HCI	x	x			
Psychology			x	x	

The perspectives of Wilkens et al. (2021) largely coincide with the results of the demonstrator. Although all the perspectives are covered, HFE is particularly concerned about the data *reliability-oriented understanding* of HCAI and the *potential-oriented understanding*.

Potential deficits of *data reliability* are mainly considered from an ethical perspective since technical artifacts are not ascribed to any moral competence; at best, they can imitate human moral behavior. Taking into account the instrumental character of AI, it is rather necessary to consider fundamental conflicts of interest that might favor immoral behavior. This, however, leaves the field of AI design.

The *potential-oriented understanding* promotes a hybrid design approach corresponding to the “complementary division of functions between humans and AI.” The mutual reinforcement of humans and HCAI appears to be suitable for satisfactorily coping with future, currently potentially unknown working requirements. It emphasizes the evolutionary principle of humans, whose behavior is imitated by intelligent machines, and that will ultimately be reflected in the precision and reliability of machine procedures.

The information science perspective on human-centered AI can be summarized mainly as *protection-oriented, potential-oriented*, and *political-oriented*. It is *protection-oriented* when it studies how information systems should be designed so that humans can easily and safely use them, and it is *potential-oriented* since it considers information systems sociotechnical environments in which humans co-construct information with the help of technology. In social settings, and especially at the workplace, the *political-oriented* perspective is also part of information science's agenda, e.g., in terms of information literacy.

An adult education perspective, which in its tradition always includes an advocacy perspective, can be contextualized as a *protection-oriented*, a *potential-oriented*, and a *policy-oriented* approach. It is *protection-oriented* when talking about qualifying workers for correct decision-making in cooperation with AI systems. It is *potential-oriented* when thinking about how to use AI systems for quality learning opportunities, e.g., in learning analytics. Finally, when reflecting on the advocacy tradition of empowering social subjects, an adult education perspective is *politically oriented*.

It is not surprising that from a HCI and psychology point of view, most perspectives by Wilkens et al. (2021) are relevant, as they touch technology and individual aspects at the same time. Interestingly, the *deficit-oriented* understanding and data *reliability-oriented* understanding perspectives are more related to HCI. So assisted tools that work through elaborated sensor technology are common to, e.g., gamified and explainable AI, which are fields of HCI.

In contrast, the *protection-oriented* understanding and *potential-oriented* understanding perspectives are closely related to psychology. Unrealistic user expectations questioning the “intelligence” of AI, In connection with loss of autonomy and trust, are prevailing psychological aspects of human-centricity for these two perspectives. Nevertheless, these areas can also be found in HCI and, depending on the degree of technical implementation, are likely to be assigned to HCI.

It is clear from Table 1 that the disciplines do not cover all of Wilkens et al. (2021) perspectives, but there are different emphases, with an imbalance existing for the first two perspectives. The article at hand shows that all disciplines are important for HCAI, with HFE functioning as an umbrella discipline for HCAI at work. This makes the call for an interdisciplinary view obvious. The following section will detail these views that are enriched by findings from a demonstrator.

### 6.2. Interdisciplinary views of human-centered AI

This section presents the different fields of action, the relevant factors of human-centered AI from different disciplines, and the interdisciplinary views of HCAI, including possible areas of collaboration. Adding insights from a demonstrator, the foundations for a synergistic human-AI symbiosis theory are revealed.

For *HFE*, human-centricity means involving humans in the design of work systems, e.g., processes or tools. The design is based on a comprehensive understanding of users, tasks, and work environments. Human-centered design aims at balanced skill and performance development as the basis of work productivity and health. *HFE* combines the human potential with technology. From a HFE perspective, human-centered AI emphasizes that the decision-making competence of humans and their intervention in technical systems in doubt are weighted higher than that of AI machines. Information science and HCI provide details and key features of how to calibrate this interaction of humans and computers. A key factor lies in aspects of designing AI systems with regard to personality issues, search behavior, and information literacy of users.

From an *adult education* perspective, the success of human-centered AI lies in providing learning opportunities across all target groups affected by AI. These learning opportunities need to adapt to learners and their individual learning habits and learning needs in terms of content and methodology instead of one-fits-all solutions. Especially marginalized learners will have to be a focus. Information science can add to this perspective with its differentiated insights on information literacy, a concept that might be helpful when implementing HCAI solutions and, up-to-date, is far away from being profoundly treated in the field of adult education with, e.g., low-skilled adults. Furthermore, HFE perspectives can help in elaborating adult learning methodologies, as HFE provides clear perspectives on workplace learning and how it adds to effective learning.

Additionally, mutual reinforcement of humans and AI, which considers ethical principles during design, is essential and is actively considered in *information science*, e.g., in aspects of usability and interaction design. Especially the more profound understanding of sociotechnical information systems and how humans interact with digital environments are essential to information science.

Finally, for *HCI and psychology*, human-centered processes and products are the foundation of human-centricity. As a result, user experience design approaches and ongoing human performance evaluation are required. Additionally, human-centered AI products are emphasized by human control (Shneiderman, 2022, p. 9). These elements should be utilized to help tackle the six grand challenges of human-centered AI: human wellbeing, responsible design of AI, privacy aspects, AI-related design and evaluation frameworks, the role of government and independent oversight, and finally, HCAI interaction in general (Garibay et al., 2023). The overlap between HFE and HCI is visible where interaction design is considered from a perspective when knowledge and experience of usability and user experience are applied. This helps, among other things, to implement technology acceptance models and, thus, to build trust and support when the overlap between HFE and HCI is consistently implemented. In addition, HFE and psychology (and partly also HCI) meet in the field of human autonomy. Furthermore, the relationship between information science and HCI is exemplified in the interaction of any kind of information and the focus on human-centered systems, again considering usability and user experience. Recognition of the importance of information literacy should be considered an important link for HCAI in this regard.

In order to validate the findings compiled here, a demonstrator is used. The project “Connect & Collect: AI-based Cloud for Interdisciplinary Networked Research and Innovation for Future Work (CoCo)” (CoCo Website, 2023) promotes the transfer of knowledge between HFE research and operational practice in companies about artificial intelligence and is funded by the German Federal Ministry of Education and Research (BMBF). To support our theory development, the CoCo project serves as a demonstrator to illustrate the interrelation between the different disciplines. The purpose of the demonstrator is to reveal the necessity of each discipline, namely HFE, psychology, HCI, information science, and adult education, to create successful HCAI implementation at work and, therefore, to show the impact and potential in society. Participants are predominantly transdisciplinary actors in labor research from science, enterprises, unions, education, and intermediaries pursuing an innovative new approach or applying best practices and have joined forces in “Regional Competence Centers for Labor Research.”

It can be derived from the demonstrator that while many companies are interested in implementing AI applications, they shy away from the research and investment effort involved in developing company-specific solutions. Instead, they aim to use proven AI applications. In this case, the importance of human-centered AI design-especially regarding learning facilitation and ethical-social compatibility-is not sufficiently applied (Pokorni et al., 2021). Undesirable consequences usually emerge only after a time delay and are rarely causally associated with AI use. An essential part of the work of the demonstrator is to increase the relevance of human-centered design of AI applications practically and systematically, which will also enhance the role of HFE experts.

At the same time, it is crucial to qualify the workers for the changes in the working society. The examples from the demonstrator show that, when implementing AI systems on a large scale, it is important to develop and establish a broad range of learning opportunities that can be upscaled to diverse target groups. Within the digital transformation, especially marginalized groups are at risk of getting lost in terms of workforce, labor markets, and in a democratic society. There is a need to focus on these marginalized groups when referring to human centeredness. Adult education providers and unions have an enormous role in this challenge because they have expertise in accessing the marginalized.

Human-centered AI is strongly related to HCI and psychology, albeit with different emphases. As HCI has evolved from a mainly technical field to an interdisciplinary profession, the same can be expected for HCAI. Nevertheless, there are critical challenges to overcome, like explainability, trustworthiness, or unrealistic user expectations. However, technology acceptance models can be used to build trust to accept AI systems and make them more human-centric, thus keeping expectations in line. Ultimately, achieving an explainable AI contributes to the mutual collaboration and interaction of humans and AI. For ethical reasons, AI's instrumental character must always be considered. Furthermore, AI can be made more accessible to stakeholders by implementing gamification, which can increase stakeholder commitment to increased engagement with human-centered AI, especially in the workplace. Finally, the demonstrator acknowledges the importance of ethical concerns by utilizing the human-in-the-loop concept, thus increasing trust.

It can be concluded that the transfer of human-centered research results into practical application is supported by an interdisciplinary approach that combines different ideas, knowledge, and work methods. By displaying the interrelationships between the disciplines, this article reveals in the following section further directions for research as well as concepts and features a (future) theory of HCAI should entail.

### 6.3. Setting foundations for a human-centered interdisciplinary AI theory: synergistic human-AI symbiosis theory (SHAST)

In order to advance the research field regarding human-centered AI, a conceptualization of a Synergistic Human-AI Symbiosis Theory (SHAST) has been started. SHAST takes into account that the optimal deployment of artificial intelligence in the workplace needs the establishment of a symbiotic relationship between humans and AI systems, drawing upon the expertise of five distinct disciplines: human factors engineering (HFE), human-computer interaction (HCI), psychology, information science, and adult education. Based on the present findings, all five disciplines appear to be necessary to successfully implement HCAI in the workplace. SHAST envisions a future where AI systems and humans collaborate synergistically to achieve unprecedented levels of productivity and wellbeing. The five disciplines presented here are predestined to contribute to the future of HCAI, as they incorporate fundamental components of human centeredness and address important fields of action for its creation and implementation.

SHAST posits that AI should be designed to augment human capabilities, foster seamless interactions, and ensure ethical practices. HFE is the foundational pillar, advocating for AI systems that enhance human potential while preserving human autonomy, decision-making, and overall work performance. HCI, another elementary bedrock, focuses on user-centric design, creating seamless and intuitive interactions between humans and AI, fostering realistic user expectations, and minimizing friction in collaborating with AI technologies that intuitively adapt to user needs and preferences. Psychology's role in SHAST revolves around cultivating user trust, achieved through transparent AI design and explainable algorithms. Information science considers the pragmatic side of information, ensuring that humans can effectively and efficiently use information systems, for example, by increasing their information literacy. Finally, adult education plays a critical part in SHAST by cultivating digital literacy and ensuring that individuals possess the skills to navigate AI-powered environments, fostering learning opportunities for a workforce to engage with AI technologies for innovation and productivity effectively, minimizing disparities, and enabling broad participation.

SHAST proposes that human-AI symbiosis can be achieved through an interplay of these disciplines, resulting in AI technologies that empower individuals, enhance collaboration, and create a sustainable and equitable future. In fact, SHAST is based on a framework that is presented in Table 2. The outcomes from theory and practice show minimum fields of action for a successful HCAI implementation.

**Table 2:** Framework with minimum fields of action for the disciplines.

**Discipline**	**Minimum fields of action**
HFE	• Balanced workload
	• Enhancing human capabilities
	• Human control
	• Social and ethical responsibility
Information science	• Pragmatics of information
	• Consider contexts and information behavior
	• Information literacy
Adult education	• Include low-skilled workers and workers' unions
	• Tailored learning and teaching in courses and the workplace
HCI and Psychology	• Technology acceptance and adoption
	• Human autonomy and control
	• Realistic user expectations
	• Explainable AI
	• Trust

If fundamental aspects from the framework of the five disciplines are missing for the implementation and application of HCAI, then this will result in severe consequences and challenges (Stephanidis et al., 2019; Garibay et al., 2023; Xu et al., 2023). More precisely, it is postulated that when a discipline is not adequately considered, there may be a failure to comply with the minimum fields of action, and implementation may not be successful.

It remains open for discussion and empirical research on which disciplines would further be needed to support the development of a widely applicable theory of human-centered AI. The article aims to convincingly present the normative, theoretical, and methodological concepts from human factors and ergonomics (HFE), psychology, human-computer interaction, information science, and adult education and why they are considered critical building blocks for HCAI and SHAST.

## 7. Conclusions for human-centered AI at work from theory and practice

In the last section, the article summarizes how it contributes to the ongoing scientific discussion on HCAI. Conclusions are drawn for HCAI at work by leveraging insights from disciplines focusing mainly on individuals (psychology), technology (HCI), work (HFE), or work context as one research field amongst others (information science and adult learning). From the fundamental disciplinary considerations and the current experiences and observations from the demonstrator, theoretical implications are derived to bring HCAI in line with today's demands of workers and companies to reflect human centeredness when dealing with the complexity of information, data, and decisions. Furthermore, the article also highlights the relevant internal logic of the individual disciplines and reveals possible mutual complementarity. It mainly argues that, although human-centered AI is a popular concept across disciplines today (Capel and Brereton, 2023), successful HCAI and its design are in strong need for an interdisciplinary approach, as all disciplines conceptualize “their humans” differently in their views and methodological approaches.

Besides the differences in detail, our comparison of the different disciplinary approaches to conceptualizing HCAI and bringing it into practice has revealed important similarities. All presented disciplines have the human at their cores — the development of human-centered AI systems is therefore deeply connected to aspects considered central to humans and that cannot be substituted with machines, such as learning and constructing information. Technology, along with its AI systems, is considered a means for human development-therefore putting the human in a superior position, which also has to be guaranteed by certain regulations to prevent AI from overruling human decision-making. The disciplines also widely agree that human-centricity can only be achieved if struggles with the use of technology are minimized. In the end, it is always a human being in a company who will use technology. Depending on the degree of how much the AI design and AI implementation process reflects the users, including their skills, trust, and their work tasks — the users will cope or fail with technology within their workplace. Hence, raising the issue of humans practically coping or failing with technology use in the workplace as a common theoretical and practical problem of human centeredness might open scientific discourse between disciplines and practice in companies (Nowotny et al., 2001).

This article is based on collaborative scholarly inquiry and a review of discipline-specific literature on the phenomenon of HCAI and joint reflection against the background of the demonstrator used in our research to study HCAI. This led to the findings and arguments in this article, which are summarized in Table 2. The article combines perspectives from five disciplines-however, we are still in the first step, i.e., conceptualization, of a five-phase model for theory building in the applied sciences (Swanson and Chermack, 2013). The need to include further or remove disciplines will become obvious further down the road of the Theory-Research-Practice Cycle that is followed and will include operationalization, confirmation, application, and refinement (Swanson and Chermack, 2013) of the concept we have come up with. Hence, this research is not finished but at the beginning of understanding what might be critical for HCAI at work.

The first results of the conceptualization stage concerned with successful HCAI implementation at work and with the common focal points of the five disciplines are the founding pillars for a Synergistic Human-AI Symbiosis Theory (SHAST), which answers the research question of what is critical for HCAI at work. It has become apparent, however, that determining the critical aspects of HCAI at work, enabling a symbiotic relationship between humans and AI systems, and describing their interplay theoretically is a complex task. Whether the challenge does not rather require investigation of more minimum fields of action, more disciplinary backgrounds like, e.g., knowledge management, economics, process management, simulation theory, operation research, philosophy, history, sociology, and many more, or a transdisciplinary approach (Defila and Di Giulio, 2018) involving all stakeholders in the research, development, and implementation process of HCAI at work remains an open question. It has been argued that HCAI and human centeredness will benefit from considering many different scientific perspectives.^1^ Furthermore, in today's world, disciplinary boundaries are fluid and increasingly blurred, or new disciplines are formed that encompass various aspects of other classical disciplines. We are aware of this, but due to a simplified discussion, we limit ourselves to a few disciplines and outline them, knowing that these boundaries are artificial.

The results from the demonstrator and our collaborative inquiry have shown that the disciplines covered in this article have many similar (and some different) concepts and methods in their portfolios that are most likely critical for successfully implementing HCAI at work. However, a broader interdisciplinary discussion and research are needed for a complete view of the topic.

## Data availability statement

The original contributions presented in the study are included in the article/supplementary material, further inquiries can be directed to the corresponding authors.

## Author contributions

AM: Conceptualization, Writing—original draft, Writing—review & editing, Funding acquisition, Supervision, Methodology, Project administration, Validation, Visualization. CB-S: Conceptualization, Writing—original draft, Writing—review & editing, Methodology, Project administration, Validation, Visualization. MB: Conceptualization, Writing—original draft, Writing—review & editing, Methodology, Project administration, Validation, Visualization. IP: Conceptualization, Writing—original draft, Writing—review & editing, Methodology, Project administration, Validation, Visualization.

## Footnotes

^1^ https://www.frontiersin.org/research-topics/50257/human-centered-ai-at-work-common-ground-in-theories-and-methods
